# Identification of a novel xanthan-binding module of a multi-modular *Cohnella* sp. xanthanase

**DOI:** 10.3389/fmicb.2024.1386552

**Published:** 2024-03-26

**Authors:** Rui Han, Melanie Baudrexl, Christina Ludwig, Oksana V. Berezina, Sergey V. Rykov, Wolfgang Liebl

**Affiliations:** ^1^Chair of Microbiology, School of Life Sciences, Technical University of Munich, Freising, Germany; ^2^Bavarian Center for Biomolecular Mass Spectrometry (BayBioMS), School of Life Sciences, Technical University of Munich, Freising, Germany; ^3^National Research Centre “Kurchatov Institute”, Moscow, Russia

**Keywords:** *Cohnella*, xanthan, xanthanase, xanthan-binding module, mobility shift, CBM66

## Abstract

A new strain of xanthan-degrading bacteria identified as *Cohnella* sp. has been isolated from a xanthan thickener for food production. The strain was able to utilize xanthan as the only carbon source and to reduce the viscosity of xanthan-containing medium during cultivation. Comparative analysis of the secretomes of *Cohnella* sp. after growth on different media led to the identification of a xanthanase designated as *Csp*Xan9, which was isolated after recombinant production in *Escherichia coli*. *Csp*Xan9 could efficiently degrade the β-1,4-glucan backbone of xanthan after previous removal of pyruvylated mannose residues from the ends of the native xanthan side chains by xanthan lyase treatment (XLT-xanthan). Compared with xanthanase from *Paenibacillus nanensis*, xanthanase *Csp*Xan9 had a different module composition at the N- and C-terminal ends. The main putative oligosaccharides released from XLT-xanthan by *Csp*Xan9 cleavage were tetrasaccharides and octasaccharides. To explore the functions of the N- and C-terminal regions of the enzyme, truncated variants lacking some of the non-catalytic modules (*Csp*Xan9-C, *Csp*Xan9-N, *Csp*Xan9-C-N) were produced. Enzyme assays with the purified deletion derivatives, which all contained the catalytic glycoside hydrolase family 9 (GH9) module, demonstrated substantially reduced specific activity on XLT-xanthan of *Csp*Xan9-C-N compared with full-length *Csp*Xan9. The C-terminal module of *Csp*Xan9 was found to represent a novel carbohydrate-binding module of family CBM66 with binding affinity for XLT-xanthan, as was shown by native affinity polyacrylamide gel electrophoresis in the presence of various polysaccharides. The only previously known binding function of a CBM66 member is exo-type binding to the non-reducing fructose ends of the β-fructan polysaccharides inulin and levan.

## Introduction

Xanthan is a natural bacterial polysaccharide secreted by *Xanthomonas* species such as the plant pathogenic *Xanthomonas campestris* (Rogovin et al., 1961; Petri, 2015). It has a β-1,4-linked glucan backbone with mannosyl-glucuronosyl-mannosyl side chains attached to the C-3 position of every second glucose residue (Jansson et al., 1975; Berezina et al., 2024). Depending on the production conditions, the inner mannosyl residues of the side chains are often acetylated while the terminal mannosyl residues are pyruvylated (Tait et al., 1986; García-Ochoa et al., 2000). Xanthan exists in a helical conformation with an order–disorder transition depending on changes in temperature and ionic strength (Bezemer et al., 1993). In addition to being a non-toxic and safe polymer, its unique properties, such as high stability over a wide range of pH and temperature, high pseudoplasticity and excellent biocompatibility have led to a wide range of applications of xanthan, for example in the manufacture of foods, pharmaceuticals and cosmetics or as a drilling fluid rheology control agent in the petroleum industry. Because of its unusual structure comprised of a cellulose-like backbone with anionic, substituted side chains arranged as ordered double-helical structures (Rinaudo and Milas, 1980; Petri, 2015), efficient xanthan degradation by microbial enzymes is challenging. So far, several bacteria capable of degrading xanthan have been described, including representatives from the mesophilic genera *Bacillus*, *Paenibacillus*, *Cellulomonas* and the thermophilic planctomycete *Thermogutta terrifontis* (Cadmus et al., 1982; Ruijssenaars et al., 1999; Liu et al., 2005; Muchová et al., 2009; Slobodkina et al., 2015).

Typically, xanthan degradation and utilization by bacteria involves extracellular xanthan lyase, which cleaves off the terminal, usually pyruvylated mannosyl residues of the side chains, and an extracellular backbone-cleaving xanthanase, as well as intracellular enzymes for deacetylation and breakdown of internalized xanthan-derived oligosaccharides (Hashimoto et al., 1998, 1999; Nankai et al., 1999, 2002). The bacterial extracellular enzymes involved in the initial steps of xanthan breakdown can also be used for xanthan modification and for the production of xanthan oligosaccharides (Berezina et al., 2024). Enzymatic side chain modification of xanthan can be accomplished by xanthan lyases (Hashimoto et al., 2003; Yang et al., 2014). Removal of the terminal mannosyl residues results in a reduction of the polymer’s viscosity (Ahlgren, 1991). Cleavage of the xanthan backbone by xanthanases can be useful for applications in the oil and gas industry to reduce the viscosity of xanthan-containing drilling fluids or for degradation of xanthan-rich polysaccharide cakes in wellbores (Tjon-Joe-Pin et al., 1997; Ghosh et al., 2019). Also, xanthanases are useful for the production of xanthan-derived oligosaccharides, which can have plant defense-eliciting, antibiotic effects (Liu et al., 2005; Qian et al., 2006; Wang et al., 2020), anti-oxidative properties (Hu et al., 2019) or prebiotic potential (Xu et al., 2021). But regarding the use of enzymes for partial breakdown of the xanthan polysaccharide backbone, the number of known enzymes is still limited.

Xanthanases (or β-d-glucanases for xanthan backbone cleavage) are usually large enzymes with molecular masses ranging from 101 kDa to 355 kDa, whereas a xanthanase from *Thermogatta terrifontis* was reported to have a size of merely 23.7 kDa (Nankai et al., 1999; Yang et al., 2019; Denisenko et al., 2021). The large xanthanases are composed of GH5 or GH9 catalytic modules and non-catalytic modules, some of which are polysaccharide recognition modules (carbohydrate-binding module, CBM), such as CBM84, which has a reported xanthan-binding function (Moroz et al., 2018; Jensen et al., 2019) and others uncharacterized (Ostrowski et al., 2022). Based on the amino acid sequence and in consequence structural similarity, there are currently 101 families of CBMs in the Carbohydrate-Active Enzymes database (CAZy)^1^ (Drula et al., 2022). Generally, CBMs are thought to have various functions which vary depending on the individual CBMs and their cognate substrates. Such functions include the increase of enzyme concentration around the saccharide substrates (Zverlov et al., 2001; Ni et al., 2023), the binding to specific regions of substrates (Boraston et al., 2001; Cuskin et al., 2012), the disruption of the structure of substrates (Giardina et al., 2001) and the adhesion to the surface of substrates (Singh et al., 2014). CBMs play important roles in biocatalytic polymer decomposition processes. Understanding the binding ability between CBMs and polysaccharide ligands could promote further applications in many fields, such as diagnostic analysis tool development, bioremediation or fiber modification (Shoseyov et al., 2006).

In this work, a strain *Cohnella* sp. capable of degrading xanthan was isolated from a spoiled xanthan thickener for food production. The strain was able to reduce the viscosity of growth media containing xanthan as the only carbon source. To determine the major players involved in xanthan utilization by the new strain, the secretome of *Cohnella* sp. cultivated in media containing different carbon sources was investigated via LC–MS/MS analysis. The resulting candidate xanthanase was expressed in *E. coli*, and different deletion variants were constructed to explore the functions of different modules of the xanthanase. In particular, the C-terminal module of the enzyme, which could be assigned to the CBM family 66, aroused our interest. The only previously known binding function of a member of the CBM66 family is exo-type binding to the non-reducing fructose ends of the β-fructan polysaccharides inulin and levan, whereas the heteropolysaccharide xanthan does not contain any fructose moieties.

## Materials and methods

### Isolation of a xanthan-degrading strain

Samples of spoiled xanthan thickener were collected at a food production plant (St. Petersburg, Russia). Xanthan-degrading bacteria were enriched by aerobic growth on mineral medium containing xanthan as the only source of carbon. The xanthan mineral medium (XMM) contained per liter 5 g xanthan, 0.05 g CaCl_2_, 0.8 g K_2_HPO_4_, 0.6 g KH_2_PO_4_, 0.5 g NaCl, 0.15 g MgSO_4_•7H_2_O, 0.6 g (NH4)_2_SO_4_, 2 mg FeSO_4_•7H_2_O, 1 mg MnCl_2_, pH 7.0. Samples of spoiled xanthan thickener were transferred into 750 mL conical flasks containing 100 mL XMM and incubated for 48 h at 30°C with shaking at 150 rpm. Aliquots of the enrichment cultures were then diluted with sterile water, spread on XMM agar plates. Colonies appearing after incubation at 30°C for 48 h were tested for their ability to reduce the viscosity of xanthan in liquid XMM. The strains liquifying xanthan in XMM in less than 24 h were subjected to molecular identification using 16S rDNA sequencing. As a result, a new xanthan-degrading strain was isolated, having about 98% 16S rDNA sequence identity with *Cohnella* species. The strain was deposited in the All-Russian Collection of Microorganisms (VKM) as *Cohnella* sp. 56 VKM B-36720. Xanthan gum used in this work was purchased from LANUCO GmbH (Hamburg, Germany), while xanthan lyase-treated xanthan (XLT-xanthan) was prepared according to a previously reported protocol (Moroz et al., 2018).

### Congo red assay

The xanthan hydrolytic activity of the *Cohnella* sp. isolate was investigated by congo red staining with a qualitative agar plate assay. For this, a thin layer of approximately 1.5–2 mm mineral medium containing 15% Lysogeny Broth (LB), 0.05% xanthan and 2% agar was poured into petri dishes after autoclaving. Overnight culture of *Cohnella* sp. 56 VKM B-36720 and *E. coli* NEB10β grown on LB was collected, then samples of culture were spotted on the surface of the xanthan plates. After incubation for 3 days at 30°C, the plates were stained with 2% congo red solution for 10 min, followed by washing with 1 M NaCl for 10 min twice. Halo appearance around the colonies on the plates indicated xanthan hydrolysis by the bacteria.

### Secretome analysis by quantitative mass spectrometry

For secretome analysis, the strain was cultured in complex and mineral media at 30°C, 180 rpm. LB medium contained per liter 10 g tryptone, 5 g yeast extract and 5 g NaCl, pH 7.0. The composition of xanthan-containing mineral medium (XMM) is described above. Glucose mineral medium (GMM) had the same composition as XMM, but xanthan was replaced by 0.5% (w/v) glucose. 2 mL culture supernatant samples from cultures grown in XMM, GMM and LB were collected by centrifugation. Three biological replicates of 25 μL supernatant were each mixed with 10 μL of LDS sample buffer (ThermoFisher Scientific, Waltham, MA, United States) and heated to 95°C for 10 min. Samples were stored at −20°C until LC–MS/MS analysis.

The in-gel trypsin digestion was performed as previously described (Shevchenko et al., 2006; Heinze et al., 2018). Briefly, 25 μL per sample were run on a Nu-PAGE™ 4–12% Bis-Tris protein gel (ThermoFisher Scientific) for approximately 1 cm. The single unseparated protein band was then excised, reduced (50 mM dithiothreitol), alkylated (55 mM chloroacetamide) and digested overnight with trypsin (Trypsin Gold, mass spectrometry grade, Promega). The obtained peptides were dried to completion and resuspended in 25 μL of 2% acetonitrile, 0.1% formic acid in HPLC grade water. Finally, 5 μL of sample was injected per mass spectrometric (MS) measurement. LC–MS/MS data acquisition of all secretome samples was performed on a Dionex Ultimate 3,000 RSLCnano system coupled to a Q-Exactive HF-X mass spectrometer (ThermoFisher Scientific). Briefly, injected peptides were delivered to a trap column (ReproSil-pur C18-AQ, 5 μm, Dr. Maisch, 20 mm × 75 μm, self-packed) at a flow rate of 5 μL/min in 0.1% formic acid (FA). After 10 min of loading, the peptides were transferred to an analytical column (ReproSil Gold C18-AQ, 3 μm, Dr. Maisch, 450 mm × 75 μm, self-packed) and separated using a 110 min gradient of 4 to 32% solvent B (0.1% FA, 5% DMSO in acetonitrile) in solvent A (0.1% FA, 5% DMSO in HPLC grade water) at a flow rate 300 nL min^−1^. The Q-Exactive HF-X mass spectrometer was operated in data dependent acquisition (DDA) and positive ionization mode. MS1 spectra (360–1,300 m/z) were recorded at a resolution of 60 k using an automatic gain control (AGC) target value of 3e6 and a maximum injection time (maxIT) of 45 ms. Up to 18 peptide precursors were selected for fragmentation. Only precursors with charge states 2 to 6 were selected and dynamic exclusion of 30 s was enabled. Peptide fragmentation was performed using higher energy collision induced dissociation (HCD) and a normalized collision energy (NCE) of 26%. The precursor isolation window width was set to 1.3 m/z. MS2 resolution was 15 k with an AGC target value of 1e5 and maxIT of 25 ms.

### Proteomic data analysis

Peptide identification and quantification were performed using the software MaxQuant (version 1.6.3.4) (Tyanova et al., 2016) with its built-in search engine Andromeda (Cox et al., 2011). MS2 spectra were either searched against the *Cohnella* sp. protein databases supplemented with common contaminants (built-in option in MaxQuant). Trypsin/P was specified as the proteolytic enzyme. Carbamidomethylated cysteine was set as the fixed modification. Oxidation of methionine and acetylation at the protein N-terminus were specified as variable modifications. Results were adjusted to a 1% false discovery rate at the peptide spectrum match (PSM) level and at the protein level employing a target-decoy approach with reversed protein sequences. The minimum peptide length was defined as 7 amino acids and the “match-between-run” functionality was disabled. The proteins differentially regulated under different conditions were further evaluated using the label-free quantification algorithm (LFQ) by Log_2_ transformation. Proteins which were identified in three replicates in at least one group (GMM, XMM and LB) were considered. The mass spectrometric raw files as well as the MaxQuant output files have been deposited to the ProteomeXchange Consortium via the PRIDE repository under the dataset identifier PXD050379.

### Genome sequence analysis and gene manipulations

For isolation of genomic DNA from *Cohnella* sp. 56 VKM B-36720, cells were disrupted using lysozyme and 1 × Tissue and Cell Lysis Solution (Biozym Scientific GmbH, Hessisch Oldendorf, Germany), where RNase A and Proteinase K were added to degrade RNA and protein, respectively. After protein removal with CTAB protein precipitation reagent (2% cetyltrimethylammonium bromide, 1% polyvinylpyrrolidone, 100 mM Tris–HCl pH 8.0, 1.4 M NaCl, 20 mM EDTA), genomic DNA was precipitated by isopropanol, dried and finally dissolved in ultrapure H_2_O. Whole genome sequence analysis was carried out by the ZIEL Core Facility Microbiome (TU Munich, Freising, Germany) via shotgun sequencing using Illumina PE150 technology on a NovaSeq platform. The sequence reads were assembled with Unicycler v4.6 and annotation was performed using the genome annotation tool Prokka 1.14.6 (Seemann, 2014) and dbCAN2 database (Zhang et al., 2018).

Amplification of the putative xanthanase gene encoding *Csp*Xan9 was performed using Q5-polymerase following the protocol from the manufacturer (New England Biolabs, Feldkirchen, Germany). All primers used in this study are listed in Supplementary Table S2. Appropriate primers were used to amplify different regions of *Csp*Xan9 in order to generate xanthanase deletion variants *Csp*Xan9-C, *Csp*Xan9-N and *Csp*Xan9-C-N. Upon amplification, gene fragments were cloned into NdeI/XhoI-linearized plamids pET24c using the Gibson Assembly Master Mix (New England Biolabs). A Strep-tag®II-encoding nucleotide sequence (TGGAGCCACCCGCAGTTCG AAAAG) was included into the primers so that the recombinantly produced xanthanase derivatives had a Strep-tag®II at the N-terminus in addition to a His-tag at the C-terminus, which is encoded on the pET24c backbone. *E. coli* DH10B competent cells were transformed with the plasmid constructs by heat-shock transformation. LB agar plates containing 50 μg mL^−1^ kanamycin were used to select transformants. After isolating recombinant plamids from overnight cultures using the protocol of the Monarch® Plasmid Miniprep Kit (New England BioLabs), the expected gene sequence and correctness of plasmid construction were confirmed by sequence analysis (Eurofins Genomics, Ebersberg, Germany).

### Enzyme production and purification

Correct recombinant plasmids were transferred into *E. coli* Rosetta2 by electroporation. Single colonies were inoculated into LB medium containing 50 μg mL^−1^ kanamycin and 34 μg mL^−1^ chloramphenicol at 37°C, 180 rpm. When the OD_600_ reached 0.5, the culture was placed on ice for 30 min and then induced by addition of 0.5 mM isopropyl-β-D-1-thiogalactopyranoside (IPTG) at 16°C, 180 rpm. Cells were harvested after 24 h by centrifugation (4°C, 12000 *g*, 30 min) and then stored at −20°C until further study.

Cell lysis was performed in buffer (50 mM MOPS, 20 mM CaCl_2_, 20 mM Imidazol, 0.3 M NaCl, pH 7.3) using sonication (settings: 50% amplitude, 0.4 s cycle, 5 min duration) with a Hielscher UP200S apparatus and the addition of 1 mg mL^−1^ lysozyme and one tablet of complete protease inhibitor (Roche Diagnostics, Rotkreuz, Switzerland). Supernatants were collected by centrifugation (4°C, 18000 *g*, 30 min) and first applied to an ÄKTApure 25 L1 FPLC system equipped with HisTrap FF columns (Cytiva Europe GmbH, Freiburg, Germany). After buffer exchange for eluted fractions from HisTrap FF columns through SephadexTM G-25 M PD-10 desalting columns (GE Healthcare, Munich, Germany), buffer W (100 mM Tris/HCl, 150 mM NaCl, 1 mM EDTA, pH 7.3) was used for further purification of *Csp*Xan9 and its deletion variants through a Strep-Tactin®XT 4Flow® high capacity cartridge (IBA Lifesciences GmbH, Göttingen, Germany). While *Cs*CBM66 was subsequently purified by a HiLoad™ 16/600 column (Cytiva Europe GmbH, Freiburg, Germany) for the second purification. Protein purity was assessed by sodium dodecyl sulfate polyarylamide gel electrophoresis (SDS-PAGE) and protein concentrations were determined by the Bradford assay using bovine serum albumin (BSA) as a standard.

### Detection of degradation products and catalytic activity

Reactions containing 5 mg mL^−1^ XLT-xanthan and 969 nM *Csp*Xan9 in 50 mM HEPES-NaOH buffer (pH 7.0) were incubated at 37°C on an orbital shaker set at 180 rpm. Samples were taken at 0 min, 5 min, 10 min, 20 min, 30 min, 1 h, 1.5 h, 2 h, 4 h, 6 h and 12 h. After denaturation at 95°C for 10 min, supernatants were collected by centrifugation and detected by high performance anion exchange chromatography with pulsed amperometric detection (HPAEC-PAD) using a Dionex ICS-6000 system equipped with a CarboPac™ PA1 column (2 mm × 250 mm) and a PA1 precolumn (2 mm × 50 mm) as described before (Angelov et al., 2017). Briefly, the reactions were diluted 10-fold before injection into the column. The eluent was 100 mM NaOH with a sodium acetate gradient, applying the following succession of conditions: 0–10 min, a linear gradient from 0 to 100 mM sodium acetate; 10–40 min, linear gradient from 100 to 800 mM sodium acetate; 40–43 min, 800 mM sodium acetate; 43–58 min, 0 mM sodium acetate. Mannose, glucuronic acid, glucose, cellobiose, cellotriose, cellotetraose, cellopentaose and cellohexaose were used as reference standards.

The specific activity of full-length *Csp*Xan9 and its three deletion variants on XLT-xanthan was measured using 2.5 mg mL^−1^ XLT-xanthan in 50 mM HEPES-NaOH buffer (pH 7.0) and appropriate enzyme concentrations (between 46.7 nM and 381.6 nM). The reactions were carried out in tubes placed into an Eppendorf thermomixer at 37°C, 1000 rpm. After appropriate periods of time, 100 μL samples were taken and cooled on ice. After centrifugation, the amounts of reducing sugars in the supernatants were quantified by 3,5-dinitrosalicylic acid (DNS) reagent followed by determination of the absorbance at 540 nm (Jain et al., 2020).

### Binding studies by native affinity polyacrylamide gel electrophoresis (NAPAGE)

NAPAGE was performed following the previously described protocol with some modifications (Meissner et al., 2000). Briefly, 6% separation gels were used for gel electrophoresis of the full-length *Csp*Xan9 and its deletion variants, while 10% separation gels were used for *Cs*CBM66. 6% separation gels were prepared by mixing 1.25 mL separation buffer (182 g L^−1^ Tris–HCl, pH 8.8) with 3 mL ultrapure water, 0.75 mL pre-mixed 30% acrylamide/bisacrylamide solution (29: 1), 30 μL of 10% (w/v) ammonium persulphate, and 15 μL of *N,N,N′,N′*-tertamethylethylenediamine. The mixture was poured between two glass plates of a Mini-PROTEAN electrophoresis system (Bio-Rad Laboratories GmbH, Feldkirchen, Germany). Soluble polysaccharides were included in the separation gels at various final concentrations as specified below. Protein samples were mixed with 4 × sample buffer (0.5 M Tris–HCl pH 6.8, 50% glycerol, 1% bromophenol blue) before loading the gels. Electrophoresis of the proteins in the polysaccharide-containing separation gels was done in electrophoresis chambers with running buffer (3.02 g L^−1^ Tris–HCl pH 8.5, 14.4 g L^−1^ glycine), after which the gels were stained by Coomassie brilliant blue R-250 solution. A NativeMark™ unstained protein standard (ThermoFisher Scientific) was used as a reference. Various polysaccharides, including xanthan, XLT-xanthan, barley beta glucan (BBG), carboxymethylcellulose (CMC), hydroxyethylcellulose (HEC), konjac glucomannan (KGM), methylcellulose (MEC), xyloglucan (XG), inulin and levan (Härer et al., 2023), were used at concentrations of 0.1 g L^−1^ - 1 g L^−1^ in the NAPAGE separation gels. The apparent dissociation constant (K_d_) for polysaccharide binding of *Cs*CBM66 was determined in the presence of 0–0.5 mg mL^−1^ XLT-xanthan in the NAPAGE gels.

## Results

### Genomic features of a xanthan-degrading strain of *Cohnella* sp.

The whole genome shotgun sequencing project of *Cohnella* sp. 56 VKM B-36720 has been deposited at DDBJ/ENA/GenBank under the accession number JAZDQE000000000. Sequence analysis of genomic DNA revealed the complete genome size was 7,167,833 bp in 41 contigs, featuring a GC content of 60.21%. To gain insights into the molecular functions, the whole genome was annotated using Prokka 1.14.6 and dbCAN2 database. Out of 5,758 protein-coding genes, 302 genes putatively encode carbohydrate-active enzymes, including 220 glycoside hydrolases (GHs), 42 glycosyltransferases (GTs), 10 polysaccharide lyases (PLs) and 19 carbohydrate esterases (CEs). Of these, 75 proteins were predicted to be secreted outside of the bacterial cell in Supplementary Table S1.

### Demonstration of xanthan-degrading activity of *Cohnella* sp.

**Figure 1:**
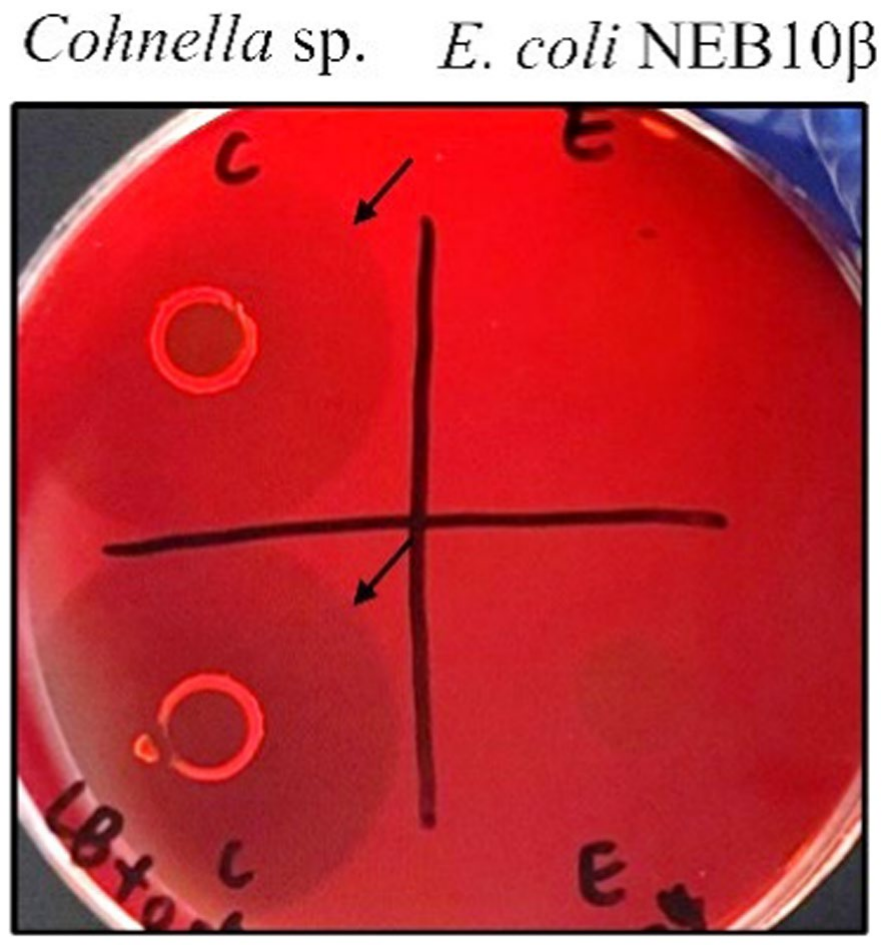
Xanthan degradation by *Cohnella* sp. strain 56 VKM B-36720 and *E. coli* NEB10β on agar plates containing xanthan visualized using Congo red staining. *Cohnella* sp. was inoculated on the left side and *E. coli* on the right side. Black arrows indicate clear halos around colonies.

Congo red dye binding to polysaccharides is a useful method for monitoring the hydrolytic activity of enzymes (Wood et al., 1988). In this study, the activity of xanthan-degrading enzymes from *Cohnella* sp. 56 VKM B-36720, which was suspected based on the ability of the isolated strain to grow with xanthan as the sole carbon source, was confirmed by the Congo red assay. *E. coli* NEB10β was used as a negative control in this experiment. After incubation for 3 days at 30°C, the strain *Cohnella* sp. and *E. coli* formed colonies on mineral medium plates containing 15% LB and 0.05% xanthan. Congo red dye staining showed clear halos around *Cohnella* sp. but not around *E. coli* colonies after washing with NaCl (Figure 1), indicating that xanthan was degraded by enzymes and utilized for bacterial growth. The fact that polymers must be degraded before uptake and utilization of breakdown products suggests that *Cohnella* sp. can secrete enzymes to degrade xanthan.

### Secretome of *Cohnella* sp. in different media

**Figure 2:**
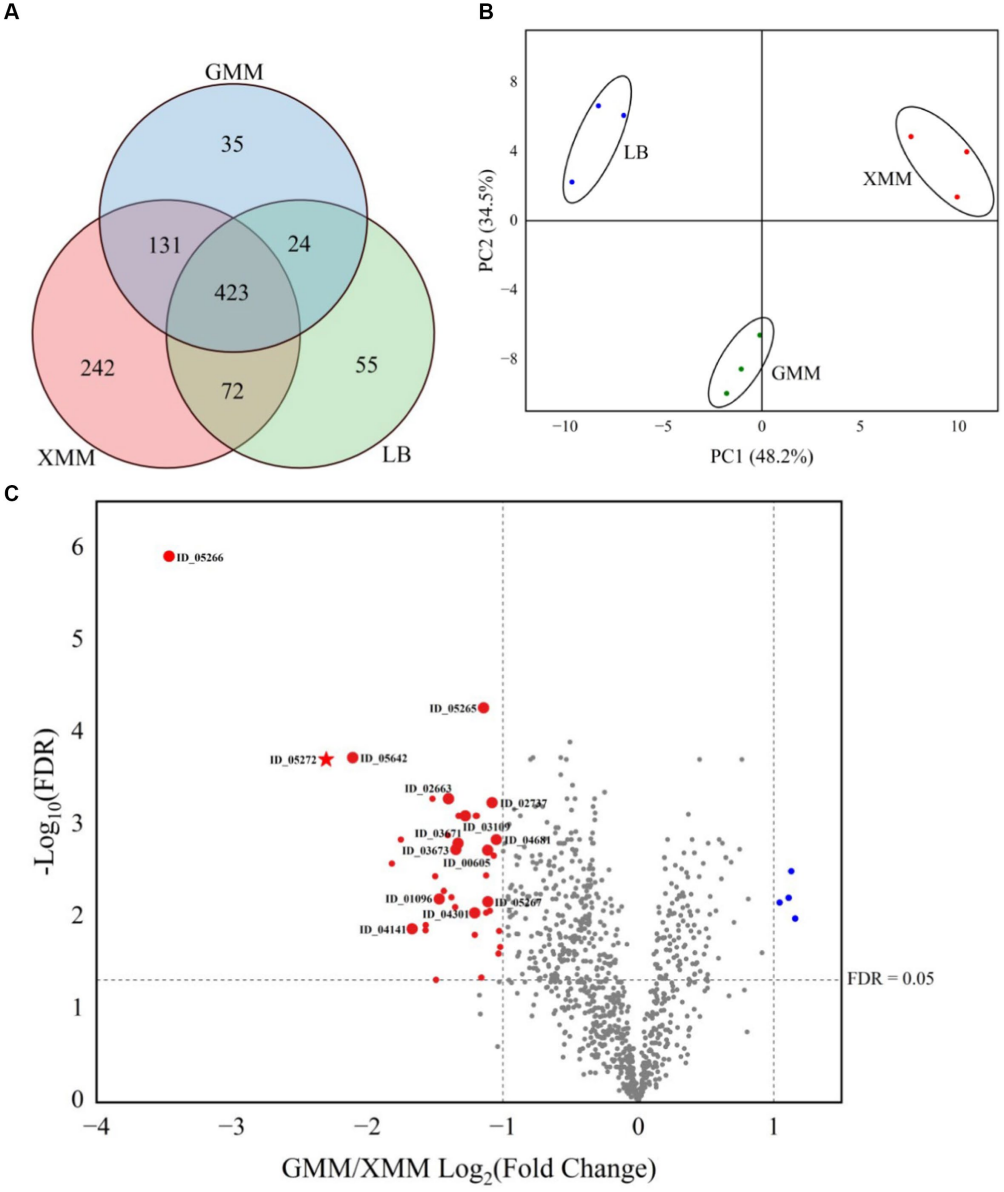
Proteomic analysis of the secretome of *Cohnella* sp. strain 56 VKM B-36720 after growth of three replicates of the strain on Lysogeny Broth (LB), Glucose minimal medium (GMM) and xanthan minimal medium (XMM). **(A)** Venn diagram of identified proteins; **(B)** PCA analysis of identified proteins; **(C)** Volcano plot comparing proteins from GMM and XMM groups. Marks in red and blue are significantly different between two groups, large red marks are unique proteins in the XMM group. A star in red represents xanthanase *Csp*Xan9 in this work.

Samples of supernatants from cultures grown on XMM, GMM and LB media (three biological replicates of cultures on each medium) were subjected to a mass sprectrometry-based proteomic analysis. Over all samples, a total of 982 proteins of *Cohnella* sp. were identified reproducibly, i.e., in at least three biological supernatants of one growth condition (Figure 2A). The largest number of unique proteins (242) were obtained from XMM, indicating that growth of the bacteria on this complex substrate leads to the production of a larger set of unique proteins compared with growth on glucose or on complex medium. A principal component analysis (PCA, Figure 2B) showed a good clustering of three replicate samples per growth condition and significant proteome changes due to different carbon sources in the growth medium. The differential expression level was analyzed by comparing the identified proteins from the GMM and XMM groups using a paired t-test, and when the false discovery rate (FDR) was ≤0.05, and fold change GMM/XMM was ≥2 or ≤ 0.5, proteins were considered to be significantly regulated by substrate. Compared with proteins from GMM, as shown in Figure 2C, four proteins (in blue) were down-regulated, while 39 proteins (in red) were up-regulated by the presence of xanthan, among which 15 proteins were detected exclusively in the XMM group (large red dots), indicating that xanthan may be an inducer for expression and/or active secretion of these unique proteins. Gene ontology (GO) functional analysis illustrated that the up-regulated proteins were mainly related to biological processes such as carbohydrate metabolic processes and organelle organization, and also related to molecular functions including hydrolase activity and carbohydrate binding. In addition, five proteins (in bold, Table 1) were predicted to be extracellular including a xanthan lyase (ID_02663) and a hypothetical protein with a GH9 module (ID_05272), typical enzymes in the xanthan degradation cascade (Nankai et al., 1999). Considering that protein (ID_05272) contains a GH9 module, it was considered as candidate enzyme putatively involved in xanthan backbone degradation. This enzyme was designated *Csp*Xan9 and studied in more detail.

**Table 1:** Unique proteins present in secretome after growth in XMM compared to GMM.

No	ID	Annotation/Blast results
1	00605	Cyclomaltodextrinase
2	**01096**	KHF37231.1|Endo-1,4-β-xylanase A precursor [*Paenibacillus* sp. P1XP2]
3	**02663**	Xanthan lyase
4	**02737**	Cyclodextrin-binding protein
5	03109	UDP-N-acetylglucosamine 4-epimerase
6	03671	WP_217594481.1|Sugar phosphate isomerase/epimerase [*Cohnella* sp. GbtcB17]
7	03673	SFB21837.1|Predicted dehydrogenase [*Cohnella* sp. OV330]
8	04141	WP_215079478.1|Hypothetical protein [*Paenibacillus polymyxa*]
9	04301	WP_277537953.1|Sporulation protein YhbH [*Cohnella rhizosphaerae*]
10	04681	BAB40444.2|α-D-mannosidase [*Bacillus* sp. GL1]
11	05265	WP_271751859.1|Hypothetical protein [*Cohnella* sp. JJ-181]
12	**05266**	WP_090114040.1|ABC transporter substrate-binding protein [*Cohnella* sp. OV330]
13	05267	Melibiose/raffinose/stachyose import permease MelC
14	**05272**	WP_158543859.1|glycoside hydrolase family 9 protein [*Cohnella* sp. OV330]
15	05642	WP_277565246.1|TIM barrel protein [*Cohnella ginsengisoli*]

The IDs of proteins putatively synthesized with an N-terminal signal peptide are in bold.

### Production and characterization of *Csp*Xan9 and deletion derivatives

**Figure 3:**
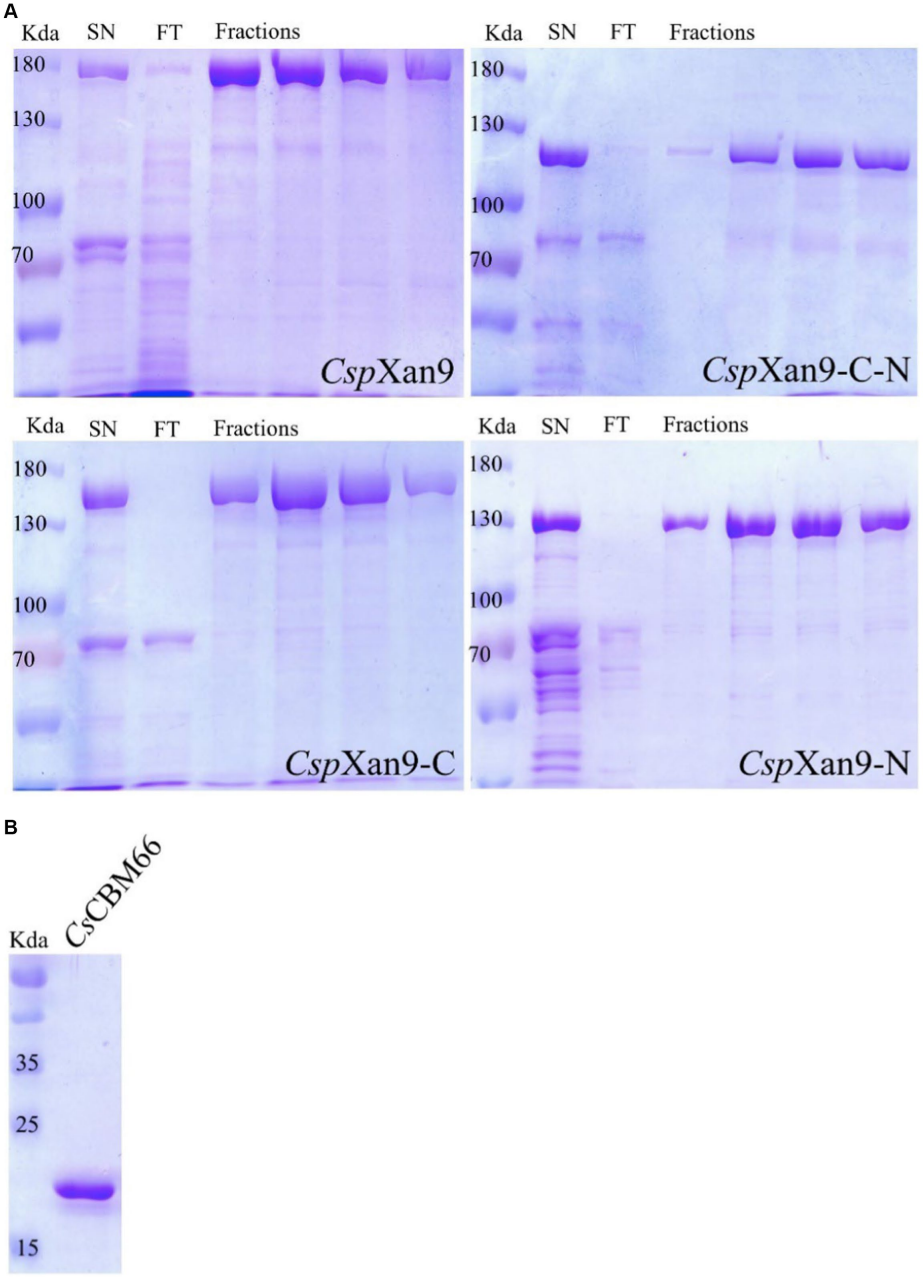
**(A)** SDS-PAGE of purification of *Cohnella* sp. strain 56 VKM B-36720 xanthanase *Csp*Xan9 and its truncated variants *Csp*Xan9-C, *Csp*Xan9-C-N and *Csp*Xan9-N. The samples loaded onto each gel represent (from left) protein molecular mass standards, fractions after elution from HisTrap FF nickel (SN), flowthrough in Strep-Tactin^®^XT column (FT) and four fractions with purified proteins after elution from Strep-Tactin^®^XT column (Fractions). **(B)** SDS-PAGE of purified *Cs*CBM66.

To investigate the function of the xanthanase candidate *Csp*Xan9, its coding sequence was amplified from the genome by PCR and assembled into the linearized vector pET24c. Production of a fusion protein with a Strep-tag®II at the N-terminus and a His-tag at the C-terminus was induced in *E. coli* Rosetta2 by the addition of 0.5 mM IPTG at a culture density of OD_600_ at 0.5, as described in the methods section. The recombinant protein was purified by immobilized metal affinity chromatography using a 5 mL HisTrap nickel column followed by a Strep-Tactin®XT cartridge. The protein purity was assessed by SDS-PAGE, which revealed abnormal electrophoretic mobility of the full-length *Csp*Xan9 with an apparent molecular mass significantly greater than the predicted mass of 148 kDa (Figure 3A).

**Figure 4:**
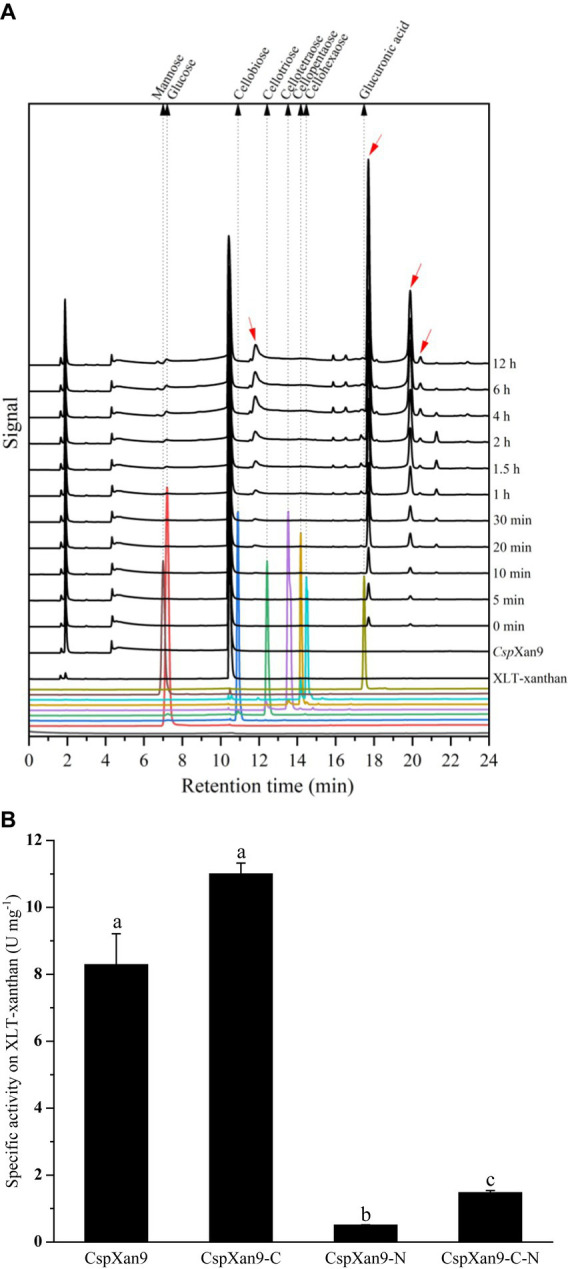
Hydrolytic activity of *Csp*Xan9 and its truncated variants. **(A)** HPAEC-PAD analysis of degradation products from XLT-xanthan by *Csp*Xan9 after 12 h incubation at 37°C, pH 7.0. Red arrows mark products that accumulated during the reaction. **(B)** Specific activity of *Csp*Xan9 and its truncated variants on XLT-xanthan. One unit of activity is defined as the amount of enzyme required to release 1 μmol glucose equivalent per minute at 37°C, pH 7.0. All tests were performed in triplicates, comparison was evaluated by one-way Dunnett’s T3 test using SPSS statistics 22 software (SPSS Inc., Chicago, IL, United States). Bars marked with different letters (a, b and c) are considered significantly different (*p* < 0.05).

Initial activity assays using different substrates revealed higher activity on xanthan lyase-treated xanthan (XLT-xanthan) than native xanthan (see Supplementary Figure S3). Therefore, all enzyme reactions were carried out with XLT-xanthan as substrate. Degradation of XLT-xanthan by purified recombinant *Csp*Xan9 was followed over time by analysis of the products released from the polysaccharide via HPAEC-PAD. Judging from the peak sizes over time, some products accumulated with increasing incubation time, and two of them with retention times of about 17.70 min and 19.88 min represented the dominant oligosaccharide products (Figure 4). Based on results reported by others for a xanthanase *Psp*Xan9 (GenBank: AXR85426.1) from *Paenibacillus nanensis* (Moroz et al., 2018), the products may represent octasaccharides and tetrasaccharides (representing xanthan fragments with four or two backbone residues, respectively, plus side chain lacking the terminal mannose). Considering the same peak patterns were produced from XLT-xanthan by *Psp*Xan9 and *Csp*Xan9 (see Supplmentary Figure S2), it can be concluded that *Csp*Xan9 also liberates tetramers and octamers, but unfortunately appropriate oligosaccharide standards are not available. Apart from the oligosaccharides, only a small amount of glucose could be detected.

**Figure 5:**
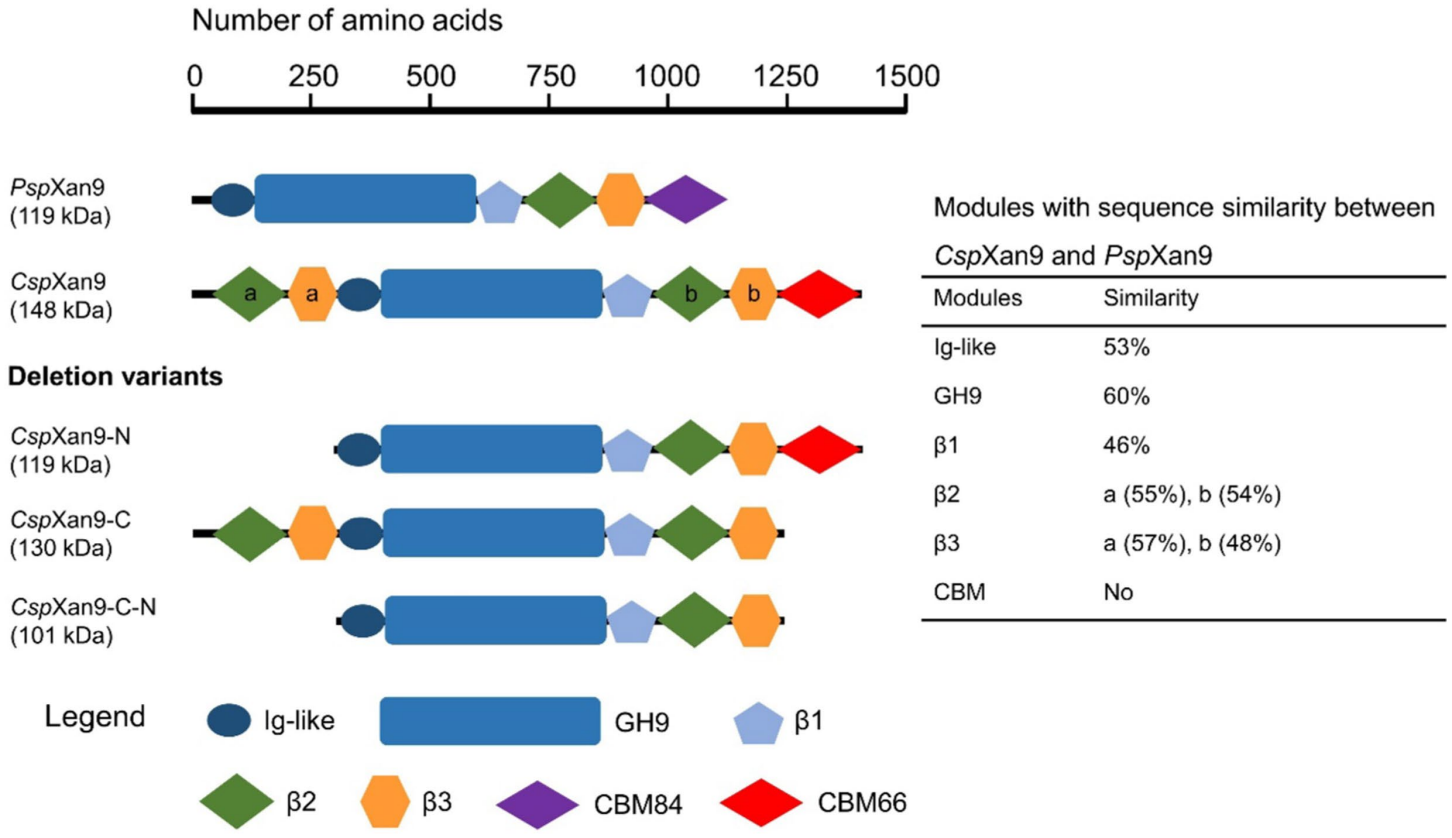
Modular structure of *Cohnella* sp. strain 56 VKM B-36720 xanthanase *Csp*Xan9 and its truncated variants. *Psp*Xan9 resembles a xanthanase from *Paenibacillus nanensis* (Moroz et al., 2018). Modules with sequence similarity are drawn with the same symbols and colors.

Large parts of *Csp*Xan9 displayed amino acid sequence similarity to the 119 kDa multi-modular xanthanase from *P. nanensis*, but *Csp*Xan9 is about 30 kDa larger due to two additional modules at its N-terminus. The full-length *Csp*Xan9 is composed of 8 modules (see Figure 5), including N-terminal β2 and β3 modules, an Ig-like module, a GH9 catalytic module, β1, β2 and β3 modules, and a C-terminal module related to carbohydrate-binding module family 66 (CBM66). BlastP analysis against the non-redundant protein sequence database available at NCBI revealed that the GH9 catalytic module of *Csp*Xan9 shared high similarity (60% amino acid sequence identity) with the catalytic module of *P. nanensis* xanthanase. Other modules (except the C-terminal module) also had more than 46% similarity with corresponding modules from the reference xanthanase (see Figure 5). The C-terminal module, however, did not show any similarity with the *P. nanensis* xanthanase, but shared up to 68.26% similarity with proteins from carbohydrate-binding module family CBM66 (see Supplementary File 1). To elucidate the functions of those modules of *Csp*Xan9 that differed from the *P. nanensis* xanthanase at the N-terminal (β2 and β3 modules) and C-terminal ends (module with unknown function similar to CBM family 66), three gene deletion variants coding for the truncated xanthanase proteins *Csp*Xan9-C, *Csp*Xan9-N and *Csp*Xan9-C-N (see Figure 5) were constructed. The truncated proteins were expressed in *E. coli* Rosetta2 under the same conditions as the full-length *Csp*Xan9. As already observed with the full-length enzyme, SDS-PAGE analysis indicated higher molecular masses than expected also for the purified *Csp*Xan9 deletion derivatives (130 kDa for *Csp*Xan9-C, 119 kDa for *Csp*Xan9-N and 101 kDa for *Csp*Xan9-C-N). Attempts to express a deletion derivative consisting only of the catalytic GH9 module of *Csp*Xan9 did not yield a catalytically active protein.

### Binding ability to different substrates

**Figure 6:**
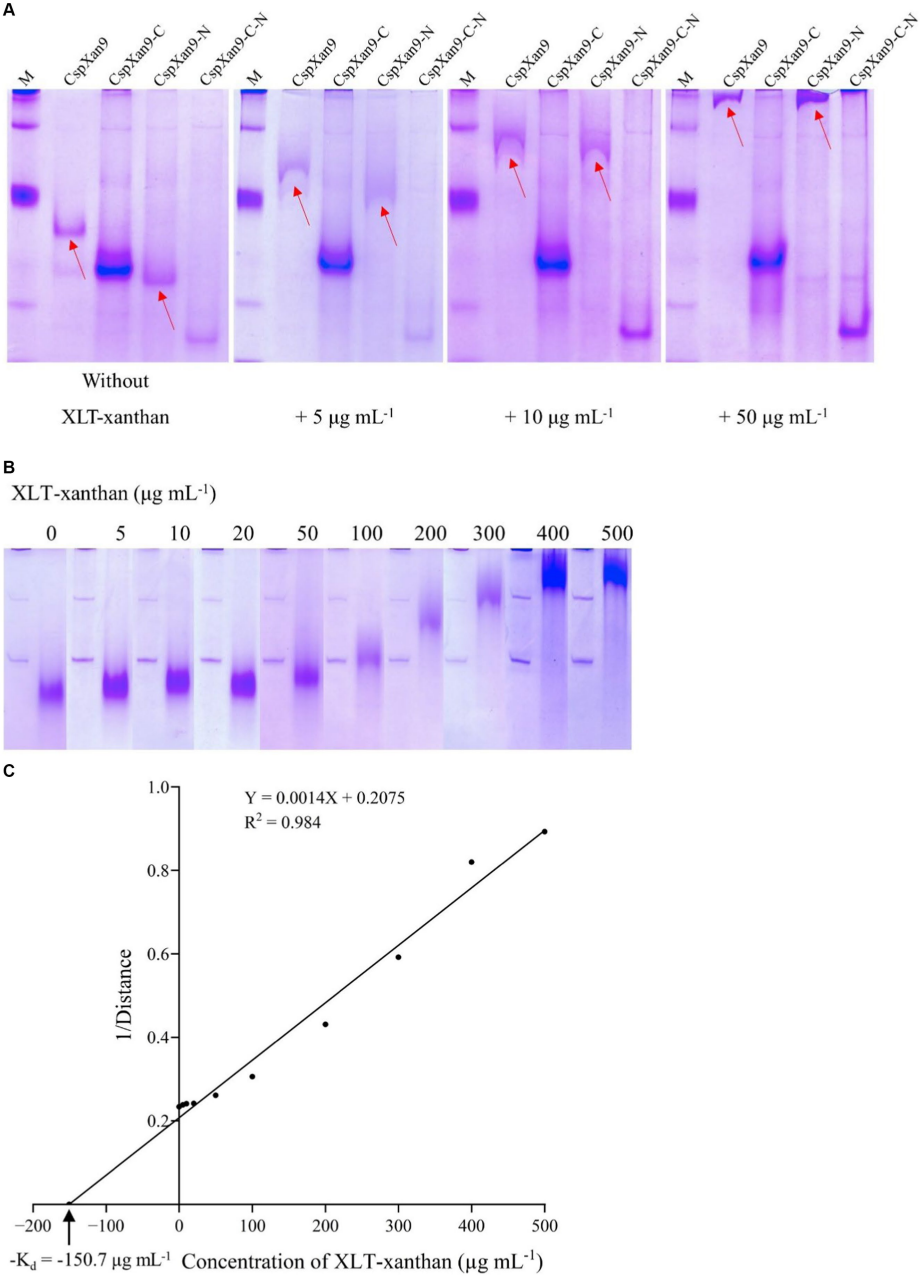
NAPAGE analysis of *Cohnella* sp. strain 56 VKM B-36720 xanthanase *Csp*Xan9 and its truncated variants. *Csp*Xan9 and its truncated variants **(A)** and *Cs*CBM66 **(B)** were separated by NAPAGE in the presence of different concentrations of XLT-xanthan. **(C)** Relationship of relative migration distance and substrate concentration.

Purified full-length *Csp*Xan9 and its deletion variants were analysed using native affinity polyacrylamide gel electrophoresis (NAPAGE) with different concentrations of XLT-xanthan included into the 6% polyacrylamide separation gel matrix (Figure 6A). Compared with a 6% polyacrylamide reference gel without added polysaccharide, significant retardation of full-length *Csp*Xan9 and *Csp*Xan9-N occured during electrophoresis in the presence of 5 μg mL^−1^ XLT-xanthan. At a 10-fold higher concentration of XLT-xanthan, the presence of the polysaccharide almost completely prevented the full-length *Csp*Xan9 and the variant with the N-terminal deletion (*Csp*Xan9-N) from entering the gel, while the variant lacking both the N-terminal β2, β3 modules and the C-terminal CBM-like module (*Csp*Xan9-C-N), as well as the variant lacking only the C-terminal CBM-like module (*Csp*Xan9-C) showed no interaction with the XLT-xanthan in the different gels.

Based on the retardation of *Csp*Xan9 and *Csp*Xan9-N in the gel, a XLT-xanthan-binding function can be assigned to the C-terminal module, which was designated as *Cs*CBM66. The only binding ability reported so far for proteins from the CBM66 family is the interaction with the terminal fructoside residue of fructans (Cuskin et al., 2012). Xanthan on the other hand does not contain any fructose moieties. In order to clarify the binding specificity of *Cs*CBM66, the 19 kDa *Cs*CBM66 module was produced in *E. coli*, and after purification (Figure 3B) the mobility of *Cs*CBM66 was studied by NAPAGE containing different substrates including various polysaccharides with a β-glucan backbone (xanthan, XLT-xanthan, BBG, CMC, HEC, MEC, XG) as well as the β-fructan polysaccharides inulin and levan. However, retardation of the mobility of *Cs*CBM66 was only observed in the presence of XLT-xanthan, but not in the gels containing fructans or other polysaccharides (Supplementary Table S3 and Supplementary Figure S1), indicating that *Cs*CBM66 specifically binds to XLT-xanthan. By analysis of the electrophoretic mobility retardation during NAPAGE in the presence of a range of different XLT-xanthan concentrations, the apparent dissociation constant (K_d_) of *Cs*CBM66 for XLT-xanthan was determined to be 150.7 μg mL^−1^ (Figure 6C).

## Discussion

### Genomic information of *Cohnella* sp. 56 VKM B-36720

Species from the genus *Cohnella* have been reported to exhibit hydrolytic activity on xylan (Yoon et al., 2007) and cellulose (Khianngam et al., 2012), suggesting that they have the enzymatic capacity to degrade complex polysaccharides from lignocellulose biomass, including hemicellulose and cellulose. The genome sizes of *Cohnella* strains typically range from 3.3 Mbp to 9.4 Mbp. The draft genome size of strain NL03-T5 was reported to be 7.44 Mbp (Wang et al., 2023), while stain *C. thermotolerans* CCUG 47242 T was determined to be 5.05 Mbp (Kim et al., 2021). The genome size of strain *Cohnella* sp. 56 VKM B-36720 (7.16 Mbp) is in the same range as strain NL03-T5, but has an unusually high GC content of 60.21% compared to other strains, although some *Cohnella* strains such as *C. ginsengisoli* DSM 18997^T^ (59.06% GC) and *C. rhizosphaerae* DSM 28161^T^ (59.48% GC) have similarly high GC contents (Simpson et al., 2023). 5.24% of the 5,758 genes of strain *Cohnella* sp. 56 VKM B-36720 encode proteins related to carbohydrate utilization, indicating that this strain could be a versatile polysaccharide utilizer. Next to being able to degrade polysaccharides from lignocellulose, we now report the ability of a *Cohnella* strain to degrade the less abundant complex polysaccharide xanthan by the ability of strain 56 VKM B-36720 to form zones of xanthan breakdown around colonies upon growth on xanthan mineral plates (Figure 1). The genome sequence indicated that a xanthan lyase, which removes the terminal (modified) mannose moiety from the trisaccharide side chains of xanthan by β-elimination (Hashimoto et al., 1998), was also present in this strain. Therefore, it is suggested that the initial steps of xanthan utilization of *Cohnella* sp. 56 VKM B-36720 may be similar to *Bacillus* sp. GL1, where five enzymes including xanthan lyase, β-d-glucanase, β-d-glucosidase, unsaturated glucuronyl hydrolase and α-d-mannosidase are responsible for complete xanthan degradation (Nankai et al., 1999). Based on the predicted presence of signal peptide-encoding sequences at their N-termini, the first two enzymes are postulated to act extracellularly, while the other enzymes are involved in the intracellular cleavage of oligosaccharides internalized by the bacterium after the extracellular oligosaccharide liberation by the combined action of xanthan lyase and β-d-glucanase.

### Exoproteomic features of *Cohnella* sp. in different media

To clarify the pathway of xanthan utilization by *Cohnella* sp. 56 VKM B-36720 and identify the key enzymes involved in the process of xanthan degradation, the secretome of the strain after growth in three different media was analyzed by mass spectrometry-based proteomics. Glucose-containing GMM and LB media were used as controls to identify unique proteins induced by xanthan during growth in XMM. Not all of the proteins identified in this way (Table 1) are predicted to be secreted proteins, which can be explained by the high sensitivity of the detection method to also detect proteins released by lysis of some cells of the cultures. Of the unique proteins found in XMM group, 10 proteins (Table 1) are presumably involved in sugar metabolism, including ID_00605, 01096, 02663, 02737, 03109, 03671, 04681, 05266, 05267 and 05272. Since xanthan has a cellulose-like backbone, enzymes from GH families 5, 6, 7, 9 and 48 theoretically could be involved in cleaving the xanthan backbone (Nguyen et al., 2018). Previously described xanthanases have been assigned to GH families 9 or 5. The GH9 xanthanase *Csp*Xan9 studied in this work (ID_05272), was identified as xanthanase candidate due to its 56% sequence similarity with *P. nanensis* xanthanase and its increased expression (4.9-fold change) in XMM group.

Protein (ID_02663) (2.6-fold change) was predicted to be an extracellular xanthan lyase, while protein (ID_04681) (2.1-fold change) presumbly represented an intracellular α-d-mannosidase. These enzymes both remove mannosyl residues. Xanthan lyase is responsible for terminal mannose removal from side chains of the xanthan structure by β-elimination, and the α-d-mannosidase (ID_04681) may represent the hydrolase that cleaves the glycosidic bond linking the inner mannosyl residues of the side chains to glucose moieties of oligosaccharides released by xanthanase-catalyzed cleavage of the β-glucan backbone. Proteins (ID_01096, 00605 and 02737), putatively a β-xylanase, a cyclomaltodextrinase and a cyclodextrin-binding protein, respectively, are also related to carbohydrate metabolism but not obviously linked to xanthan degradation, but rather to degradation of the hemicellulose xylan and of cyclodextrins, which are oligosaccharides derived from starch. During growth on xanthan, the provision of transport systems for the uptake of xanthan breakdown products can be expected (Sun et al., 2019). Indeed, further secretome proteins induced during growth on xanthan included the putative transport proteins ID_05266 and 05267. The extracellular protein (ID_05266), annotated as ABC transporter substrate-binding protein, had a strongly increased expression level (11-fold change) in the xanthan medium. Protein (ID_05267) expression increased only 2.1-fold. This protein is annotated as melibiose/raffinose/stachyose import permease MelC, but annotation tool-based prediction of the substrate specificity of permeases is error-prone. Both putative transport proteins (ID_05266 and 05267) are encoded in the genome in close proximity to the xanthanase (ID_05272), suggesting their possible role in the uptake of xanthan degradation products. Still, conclusively linking these proteins to the uptake of xanthan breakdown products requires further experimental work.

### Enzymatic activity of *Csp*Xan9

After enzyme production and purification, SDS-PAGE revealed that purified *Csp*Xan9 showed a higher molecular mass than its predicted mass based on amino acids composition, which may have to do with its content of acidic amino acids. Guan et al. found that the difference between the expected and displayed molecular mass of acidic proteins in SDS-PAGE was correlated linearly with the percentage of acidic amino acids residues (Guan et al., 2015). Although xanthanase *Csp*Xan9, an acidic protein with a predicted isoelectric point of 4.59 and the composition of 10.7% aspartate and glutamate, did not fit this reported equation, the irregular migration in SDS-PAGE might be influenced by this factor. Preliminary experiments indicated that *Csp*Xan9 had a higher hydrolytic activity on XLT-xanthan than on native xanthan, so XLT-xanthan was chosen as the substrate for most enzyme assays of the full-length enzyme and its truncated derivatives in this study. In comparison, the GH5 glycoside hydrolase *Ru*GH5a, isolated from the family Ruminococcaceae, was able to hydrolyze native xanthan and XLT-xanthan with comparable specificity (kcat/Km = 9.48 vs. 22.69) by cleaving the glycosidic bond of the non-branching glucose residues (Ostrowski et al., 2022). Besides, an endo-type GH9 xanthanase from *Microbacterium* sp. XT11 also showed hydrolytic activity on these two different xanthan substrates (Yang et al., 2019). Another endo-xanthanase containing a GH9 catalytic module from *Paenibacillus nanensis*, like *Csp*Xan9 showed its highest specific activity on XLT-xanthan, but also demonstrated weak hydrolytic activity on native xanthan (Moroz et al., 2018). Compared with *P. nanensis* xanthanase (GenBank: AXR85426.1), *Csp*Xan9 has different modules at the N- and C-terminal ends (see Figure 5). Enzymatic activity assays showed a significantly lower hydrolytic activity of the N- and C-terminally truncated enzyme *Csp*Xan9-C-N on XLT-xanthan (1.47 ± 0.07 U mg^−1^) compared with full-length *Csp*Xan9 (8.28 ± 0.93 U mg^−1^). Deletion of the two N-terminal modules β2 and β3 (*Csp*Xan9-N) resulted in an about 17-fold decrease in enzymatic activity from 8.28 ± 0.93 U mg^−1^ to 0.50 ± 0.01 U mg^−1^. On the other hand, the absence of C-terminal CBM module (*Csp*Xan9-C) led to a slightly increased enzymatic activity (11.00 ± 0.33 U mg^−1^), which seems to indicate a negative effect of the CBM on the catalytic module (Boraston et al., 2004), but this was statistically not significant.

The results of analysis of the degradation products released from XLT-xanthan by *Csp*Xan9 by HPAEC-PAD showed that only a small amount of glucose was released from the substrate, indicating that *Csp*Xan9 might have a weak exo-acting activity. There were also other products (red arrows in Figure 4A), whose amounts increased with increasing incubation time, which could not be identified by comparison with available reference sugars. The peak areas of two main products with elution times at 17.70 min and 19.88 min increased with the incubation time of the enzyme reaction. LS-ESI-MS analysis revealed that *P. nanensis* xanthanase could cleave XLT-xanthan after two or four backbone glucose residues, mainly leading to tetra- and octasaccharides as main products (Moroz et al., 2018), tetrasaccharides being the repeating units of XLT-xanthan (see Supplementary Figure S4). Based on the observation that the products released by *Csp*Xan9 displayed a similar peak pattern in HPAEC-PAD as those of *P. nanensis* xanthanase *Psp*Xan9, and the reported end products non- and monacetylated tetrasaccharides would be present in one peak in mobile phase 100 mM NaOH of chromatography, we presume that *Csp*Xan9 has a similar cleavage specificity as *Psp*Xan9, which leads to tetra- and octasaccharides after the enzyme reaction, but the exact degradation products by *Csp*Xan9 still require further investigation.

### Function and substrate specificity of *Cs*CBM66

NAPAGE analysis with 6% non-denaturing polyacrylamide gels containing XLT-xanthan showed mobility retardation of *Csp*Xan9 and *Csp*Xan9-N, which both contain the C-terminal module of *Csp*Xan9. In contrast, the electrophoretic mobility of all *Csp*Xan9 derivatives lacking the C-terminal module, i. e. *Csp*Xan9-C and *Csp*Xan9-C-N, was not affected by XLT-xanthan (Figure 6A). Thus the NAPAGE experiments clearly demonstrated that the C-terminal module has a function in binding to XLT-xanthan. Recombinant expression of the isolated module *Cs*CBM66 and NAPAGE analysis in the presence of increasing concentrations of XLT-xanthan (Figures 3B, 6B) comfirmed its function as a XLT-xanthan-binding module. To date, the known xanthan-binding modules (from *Paenibacillus nanensis* and *Microbacterium* sp. XT11) are mainly categorized into the CBM84 family, which does not share sequence similarity with *Cs*CBM66. The C-terminal module of *Csp*Xan9, now designated as *Cs*CBM66, is a member of the CBM66 family, whose representatives can be found mainly in the bacterial phyla Bacillota, Actinomycetota, Pseudomonadota and Bacteroidota, but also in some fungi (see www.cazy.org). Other CBM66 modules with sequence similarity to *Cs*CBM66 are appended to enzymes with various functions such as pectate lyase, levanase, arabinanase and others from GH43 and GH32 (see Supplementary File 1). However, the substrate binding specificities of these orthologous CBM66 modules have not been characterized. The only member of the CBM66 family characterized so far was reported to bind the terminal fructoside residue of fructans (Cuskin et al., 2012). In contrast, *Cs*CBM66 showed no interaction with levan and inulin in NAPAGE analysis.

Of various polysaccharides with a β-glucan backbone polymerized into NAPAGE gels, i. e native xanthan, XLT-xanthan, BBG, CMC, HEC, MEC, and XG, *Cs*CBM66 displayed binding affinity exclusively for XLT-xanthan, which demonstrates that the β-glucan backbone was not the main structural feature that determined polysaccharide binding. Rather, the two-moiety side chain of XLT-xanthan [unsaturated d-glucuronic acid-(1 → 2)-α-d-(*O*6-acetyl-)mannose, linked to *O*3 of every second backbone glucose residue] appears to be of crucial importance for binding of *Cs*CBM66, while an additional (pyruvylated) mannose on the side chain, as found in native xanthan, prevents binding of the CBM, since *Cs*CBM66 did not show the same electrophoretic retardation in the NAPAGE gels containing xanthan as it did in the presence of XLT-xanthan. Enzymatic activity of *Csp*Xan9 and *Csp*Xan9-C on different ꞵ-glucan substrates revealed the strongest activity on XLT-xanthan (see Supplementary Figure S3), indicating that the binding specificity of *Cs*CBM66 for XLT-xanthan largely corresponds to the cleavage specificity of the catalytic GH9 module of *Csp*Xan9, which preferentially cleaves XLT-xanthan, although the catalytic module hydrolyzes the β-glucan backbone which does not decisively contribute to *Cs*CBM66 binding, as mentioned above. However, *Csp*Xan9 displayed weak activity towards native xanthan while in contrast no binding affinity was observed between *Cs*CBM66 and native xanthan, which points to differences in substrate specificity properties between the catalytic GH9 module of *Csp*Xan9 and its C-terminal CBM module. Differences in substrate specificity between catalytic and binding modules of the same enzyme have been reported before. In some cases, CBMs and catalytic modules of multi-modular enzymes can even have completely different substrate specificities. Two examples are the xylanase XynA of *Thermotoga maritima* that contains two cellulose-binding CBMs (Winterhalter et al., 1995), and the exo-β-d-glucosaminidase CsxA whose CBM35 module interacts with a surface polysaccharide of the cell wall of *Amycolatopsis orientalis,* while its catalytic module was active on the fungal polysaccharide chitosan (Montanier et al., 2009). For *Csp*Xan9, on the other hand, both the catalytic module and the CBM recognize XLT-xanthan, albeit apparently different sub-structures thereof.

In conclusion, the work reported here contributes to the elucidation of the functions of the catalytic and non-catalytic modules of a new xanthanase *Csp*Xan9 secreted by the xanthan-utilizing strain *Cohnella* sp. 56 VKM B-36720 during growth on xanthan as the carbon source. As reported for other related xanthanolytic enzymes, the GH9 catalytic module of the enzyme predominantly cleaves XLT-xanthan, a xanthan derivative devoid of the (pyruvylated) mannose residues at the side chain termini and only displays low activity with native xanthan. Interestingly, our work shows that the C-terminal module of xanthanase *Csp*Xan9, *Cs*CBM66, has binding affinity for XLT-xanthan and that the side chain of xanthan after removal of the terminal pyruvylated mannose residue, rather than the polysaccharide’s β-glucan backbone, is of central importance for the binding specificity of this CBM. The binding affinity of *Cs*CBM66 for XLT-xanthan adds a novel binding specificity to the CBM66 family. These findings have potential applications of enzyme modification for xanthan, as combinations of the binding module *Cs*CBM66 and catalytic modules for the cellulose-like backbone from GH5, GH9 or other glycoside hydrolase families could lead to the design of highly efficient enzymes (Ni et al., 2023).

## Data availability statement

The datasets presented in this study can be found in online repositories. The names of the repository/repositories and accession number(s) can be found in the article/Supplementary material.

## Author contributions

RH: Data curation, Formal Analysis, Investigation, Methodology, Software, Visualization, Writing – original draft, Writing – review & editing. MB: Methodology, Project administration, Supervision, Writing – review & editing. CL: Data curation, Investigation, Visualization, Methodology, Writing – original draft. OB: Conceptualization, Methodology, Writing – original draft. SR: Conceptualization, Methodology, Writing – review & editing. WL: Conceptualization, Methodology, Project administration, Supervision, Writing – review & editing.

## Footnotes

^1^ www.cazy.org
