# Supplementary material for: Identification of a novel xanthan-binding module of a multi-modular Cohnella sp. xanthanase
Source: Front Microbiol. 2024 Mar 26;15:1386552. doi: 10.3389/fmicb.2024.1386552 (PMC11002231; doi:10.3389/fmicb.2024.1386552)
Supplement: Supplementary file 2 [file Data_Sheet_1.docx]

**Supplementary information**

| **Table S1. Putative extracellular carbohydrate-active enzymes encoded in the genome of *Cohnella* sp. 56 VKM B-36720 by dbCAN2 database** (Zhang et al., 2018)**.** | | | |
| --- | --- | --- | --- |
| No. | ID | Signalp | CAZy-Annotations |
| 1 | 00065 | Y(1-43) | GH39 |
| 2 | 00108 | Y(1-29) | GT2 |
| 3 | 00178 | Y(1-26) | GH43_32+CBM66 |
| 4 | 00333 | Y(1-26) | CBM6 |
| 5 | 00375 | Y(1-23) | GH87+CBM32+CBM35 |
| 6 | 00399 | Y(1-32) | CBM35 |
| 7 | 00402 | Y(1-37) | CBM35 |
| 8 | 00414 | Y(1-33) | GH26+CBM32 |
| 9 | 00424 | Y(1-26) | GH26+CBM23 |
| 10 | 00604 | Y(1-29) | GH13_36 |
| 11 | 00639 | Y(1-28) | GH16_3+CBM32+CBM4+CBM54 |
| 12 | 00641 | Y(1-42) | GH81+CBM32 |
| 13 | 00778 | Y(1-26) | GH27+CBM35+CBM61 |
| 14 | 00924 | Y(1-36) | GH3 |
| 15 | 00966 | Y(1-37) | GH87+CBM32+CBM35 |
| 16 | 01070 | Y(1-33) | GH39+CBM9 |
| 17 | 01096 | Y(1-33) | GH5_35 |
| 18 | 01100 | Y(1-36) | GH43_16+CBM6 |
| 19 | 01111 | Y(1-35) | GH10 |
| 20 | 01177 | Y(1-36) | GH87+CBM32+CBM35 |
| 21 | 01197 | Y(1-34) | GH18 |
| 22 | 01326 | Y(1-32) | GH26+CBM23+CBM27+CBM54 |
| 23 | 01353 | Y(1-25) | GH126 |
| 24 | 01731 | Y(1-29) | GH18 |
| 25 | 01851 | Y(1-31) | CBM57 |
| 26 | 02084 | Y(1-38) | GH13_39+CBM20+CBM34 |
| 27 | 02408 | Y(1-36) | GH26+CBM23 |
| 28 | 02409 | Y(1-29) | GH26+CBM32 |
| 29 | 02426 | Y(1-40) | GH16 |
| 30 | 02427 | Y(1-40) | GH16 |
| 31 | 02458 | Y(1-37) | GH3+CBM6 |
| 32 | 02481 | Y(1-21) | CBM9 |
| 33 | 02488 | Y(1-35) | GH136 |
| 34 | 02567 | Y(1-33) | GH3+GH30_3+CBM6 |
| 35 | 02591 | Y(1-29) | GH64+CBM56 |
| 36 | 02598 | Y(1-29) | GH16_3+CBM4+CBM54 |
| 37 | 02605 | Y(1-29) | GH11 |
| 38 | 02663 | Y(1-31) | PL8 |
| 39 | 02740 | Y(1-28) | GH141 |
| 40 | 02921 | Y(1-31) | GH5_2+CBM3 |
| 41 | 02937 | Y(1-32) | GH53+CBM61 |
| 42 | 02940 | Y(1-31) | GH5_2+CBM17+CBM28 |
| 43 | 03037 | Y(1-26) | GH87+CBM35 |
| 44 | 03038 | Y(1-29) | GT39 |
| 45 | 03279 | Y(1-31) | CE4 |
| 46 | 03646 | Y(1-27) | GH26+CBM13+CBM35 |
| 47 | 03661 | Y(1-33) | GH26+CBM35 |
| 48 | 03925 | Y(1-35) | GH74+CBM66+CBM9 |
| 49 | 03966 | Y(1-31) | GH10+CBM22+CBM9+CE4 |
| 50 | 03987 | Y(1-32) | GH30+CBM9 |
| 51 | 04044 | Y(1-31) | CBM32 |
| 52 | 04045 | Y(1-32) | CE2+CBM32 |
| 53 | 04350 | Y(1-29) | GH16_21 |
| 54 | 04356 | Y(1-28) | GH16_3+CBM4+CBM54+CBM6 |
| 55 | 04375 | Y(1-29) | CBM32 |
| 56 | 04496 | Y(1-32) | CBM35 |
| 57 | 04528 | Y(1-32) | GH50 |
| 58 | 04625 | Y(1-33) | GH30_3+CBM61+CBM6 |
| 59 | 04657 | Y(1-33) | GH93 |
| 60 | 04664 | Y(1-25) | GH93+CBM66 |
| 61 | 04793 | Y(1-23) | PL14_3 |
| 62 | 04802 | Y(1-34) | CE4 |
| 63 | 04955 | Y(1-47) | GH18 |
| 64 | 05106 | Y(1-40) | CBM51 |
| 65 | 05115 | Y(1-36) | GH136 |
| 66 | 05181 | Y(1-22) | GH136 |
| 67 | 05198 | Y(1-35) | GH9 |
| 68 | 05272 | Y(1-37) | GH9 |
| 69 | 05324 | Y(1-36) | GH44+CBM8 |
| 70 | 05337 | Y(1-29) | CBM54 |
| 71 | 05507 | Y(1-25) | GH26+CBM59 |
| 72 | 05585 | Y(1-26) | GH87+CBM35 |
| 73 | 05666 | Y(1-32) | GH16 |
| 74 | 05669 | Y(1-31) | GH16 |
| 75 | 05700 | Y(1-40) | GH43_24+CBM32 |

| **Table S2. Primers used for gene modifications** | | |
| --- | --- | --- |
| Primer | Sequence (5‘-3‘) | Amplification |
| 616V | AGAGTTTGATYMTGGCTC | 16S rDNA |
| 630R | CAKAAAGGAGGTGATCC |  |
| 10.C.GH9-F2 | CTTTAAGAAGGAGATATACAATGCCGCTTGTGTACGACGATTTC | *Csp*Xan9 |
| 10.C.GH9-R2 | AGTGGTGGTGGTGGTGGTGCCGCTTGACGGTCACATTG |  |
| NT StrepII-F | CCACCCGCAGTTCGAAAAGTCCGCCCCGCTTGTGTACGACGATT |  |
| NT StrepII-R | CTTTTCGAACTGCGGGTGGCTCCACATTGTATATCTCCTTCTTAA |  |
| #10-NT-CBM-F | GTCGTCGTCAGCCAGGCC | *Csp*Xan9-C-N |
| #10-NT-CBM-R | GTCGTCGGCCAGCAGCGT |  |
| pET24c-NT-Strep-F | TGCTGGCCGACGACTCCGCCCACCACCACCACCACCACTG |  |
| pET24c-CT-6his-R | CGGCCTGGCTGACGACGACGGCGGACTTTTCGAACTGCG |  |
| #10-CBM-F | TCCGCCCACCACCACCACCACCACTG | *Csp*Xan9-C |
| #10-CBM-R | GGTGGTGGTGGGCGGAGTCGTCGGCCAGCAGCGTC |  |
| #10-NT-F | AGCCAGGCCGGCTACAATGC | *Csp*Xan9-N |
| #10-NT-R | CCGGCCTGGCTGACGACGACGGCGGACTTTTCGAACTGCG |  |
| CBM66-F | CTTTAAGAAGGAGATATACAATGGCCGACGACTTCGAGGA | *Cs*CBM66 |
| CBM66-R | GGTGGTGGTGGTGGTGCGCGGACCGCTTGACGGTCACATT |  |
| pET24c-F1 | CCGAAACAAGCGCTCATGAG | Sequencing primer |
| pET24c-R1 | GAAGGGAAGAAAGCGAAAGG |  |

| **Table S3. Soluble polysaccharides applied in NAPAGE** | |
| --- | --- |
| Substrates | Structure |
| Xanthan | β-1,4-d-glucose with α-d-mannose-(2→1)-β-d-glucuronic acid-(4→1)-β-d-mannose side chain |
| XLT-xanthan | β-1,4-d-glucose with α-d-mannose-(2→1)-β-d-glucuronic acid side chain |
| Inulin | β-1,2-linked d-fructose polysaccharide |
| Levan | β-2,6-linked d-fructose polysaccharide |
| Barley beta glucan (BBG) | β-1,3/1,4-linked d-glucose polysaccharide |
| Carboxymethylcellulose (CMC) | β-1,4-linked d-glucose polysaccharide with carboxymethyl substituents |
| Hydroxyethylcellulose (HEC) | β-1,4-linked d-glucose polysaccharide with hydroxyethyl substituents |
| Methylcellulose (MEC) | β-1,4-linked d-glucose polysaccharide with methyl substituents |
| Xyloglucan (XG) | β-1,4-linked d-glucose polysaccharide with 1,6-linked xylose side chains |
| Konjac Glucomannan (KGM) | β-1,4-linked d-glucose and d-mannose polysaccharide |


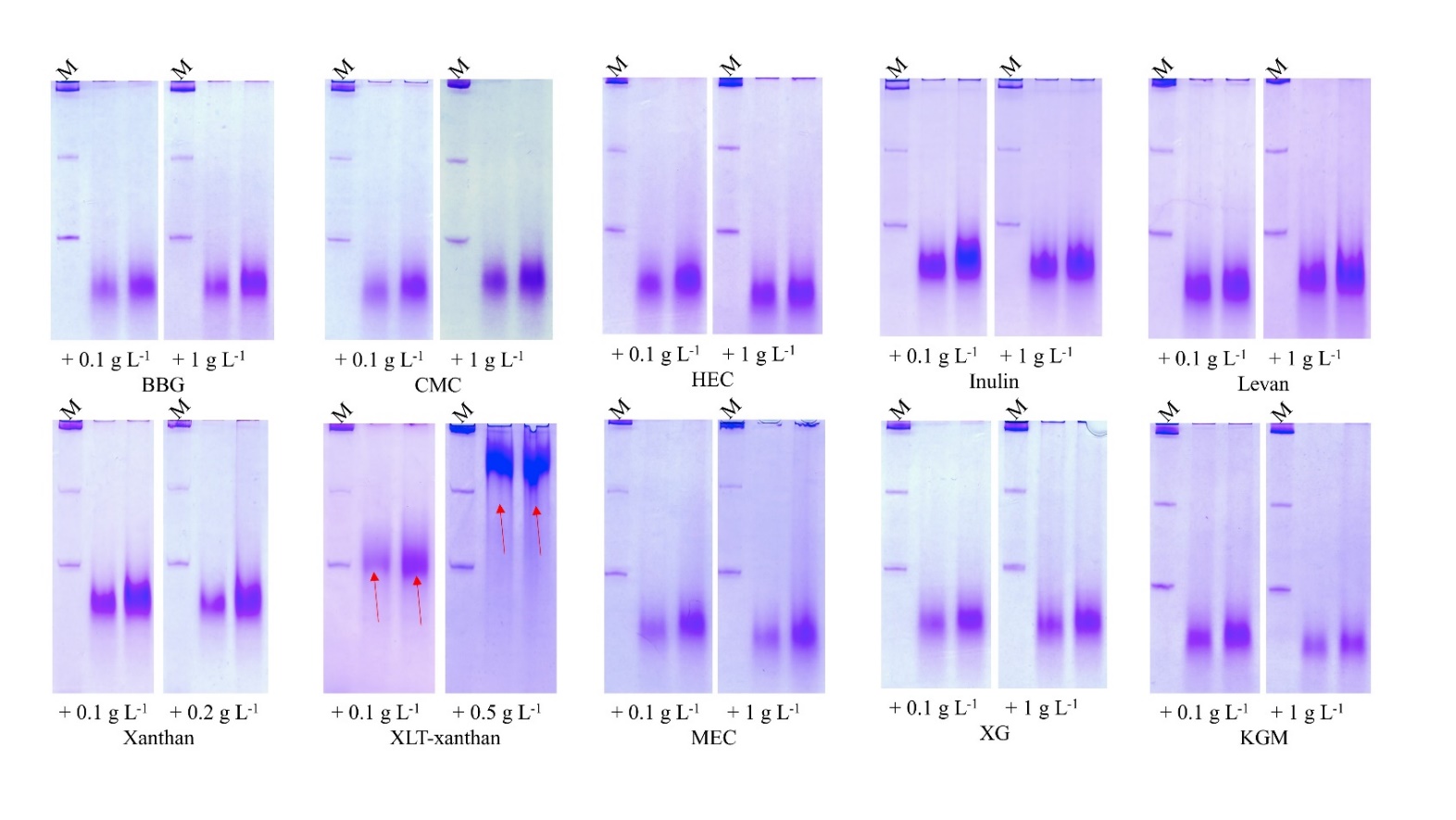


**Fig.S1 NAPAGE analysis of *Cs*CBM66 from *Cohnella* sp. strain 56 VKM B-36720 xanthanase.** The samples loaded onto each gel represent (from left) are a NativeMark™ unstained protein standard, 2 µL and 3 µL purified *Cs*CBM66 in the presence of different concentrations of various substrates as mentioned in Table S3. A significant mobility shift was only observed if XLT-xanthan was included in the NAPAGE gels.

**Fig.S2 HPAEC-PAD analysis of degradation products from XLT-xanthan by *Cohnella* sp. xanthanase *Csp*Xan9 and *Paenibacillus nanensis* xanthanase** ***Psp*Xan9.** Black arrows mark main products after enzyme reaction.

**
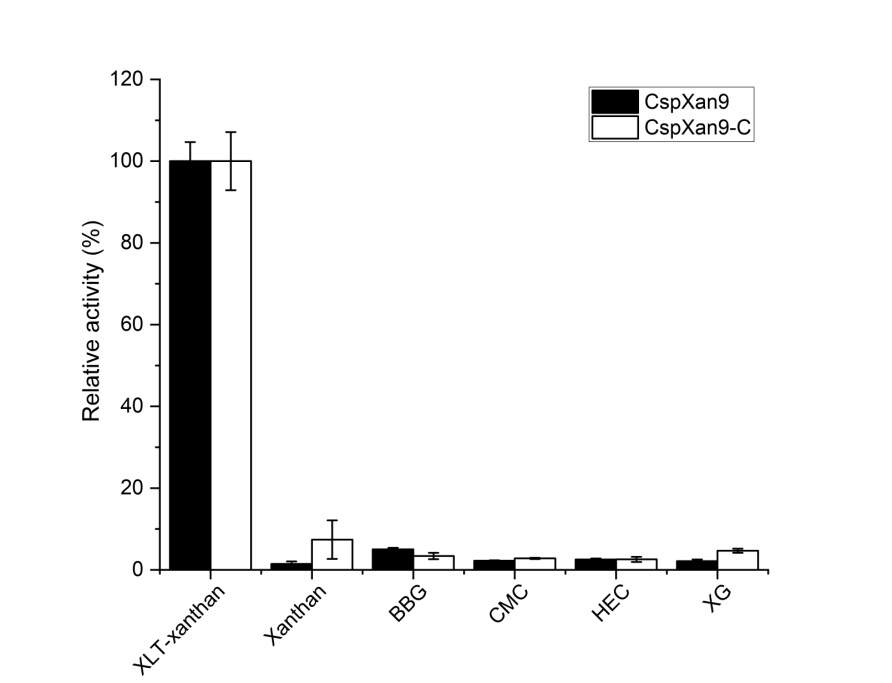
**

**Fig.S3 Relative activity of *Csp*Xan9 and *Csp*Xan9-C on different** β**-glucan substrates.** XLT-xanthan: xanthan lyase-treated xanthan, BBG: barley beta glucan, CMC: carboxymethylcellulose, HEC: hydroxyethylcellulose, XG: xyloglucan.


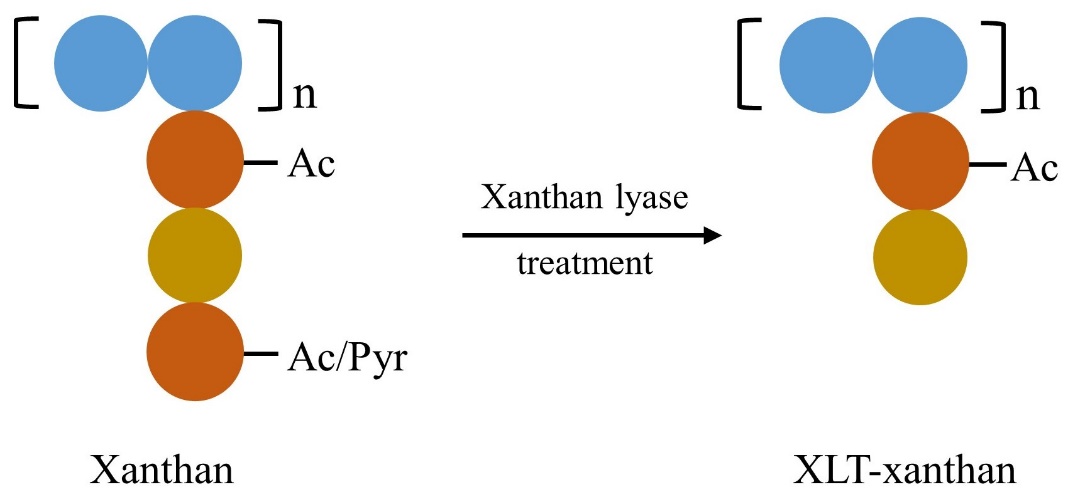


**Fig.S4 Repeating units of xanthan and XLT-xanthan**. Blue: glucose, orange: mannose, gold: glucuronic acid, Ac: acetyl group, Pyr: pyruvyl group.
